# Complete remission of metastatic osteosarcoma using combined modality therapy: a retrospective analysis of unselected patients in China

**DOI:** 10.1186/s12885-021-08071-5

**Published:** 2021-03-31

**Authors:** Lu Xie, Jie Xu, Xiaowei Li, Zuli Zhou, Hongqing Zhuang, Xin Sun, Kuisheng Liu, Xingyu Liu, Kunkun Sun, Yiming Wu, Jin Gu, Wei Guo

**Affiliations:** 1 Musculoskeletal Tumor Center, Peking University People’s Hospital, No. 11 Xizhimen South Street, Xicheng District, Beijing, PR China; 2 Department of Thoracic Surgery, Peking University People’s Hospital, No. 11 Xizhimen South Street, Xicheng District, Beijing, PR China; 3 Department of Radiotherapy, Peking University Third Hospital, No. 49 North Garden Rd., Haidian District, Beijing, PR China; 4 Pathology Department, Peking University People’s Hospital, No. 11 Xizhimen South Street, Xicheng District, Beijing, PR China; 5 Endocrinology Department, Peking University Shougang Hospital, No. 9 Jinyuanzhuang Road, Shijingshan District, Beijing, PR China; 6 Surgical Oncology, Peking University Shougang Hospital, No. 9 Jinyuanzhuang Road, Shijingshan District, Beijing, PR China

**Keywords:** Osteosarcoma, Pulmonary metastasis, Hypofractionated radiotherapy, Event-free survival, Radiation pneumonitis

## Abstract

**Background:**

Complete surgical remission (CSR) is the best predictor of overall survival (OS) for patients with metastatic osteosarcoma. However, metastasectomy has not been widely implemented in China in the last decade due to various factors, and instead, most physicians choose hypofractionated radiotherapy to treat pulmonary lesions. This study aimed to retrospectively evaluate the outcomes of different local treatments for pulmonary lesions and identify the best local therapy strategies for these patients.

**Methods:**

We reviewed the clinical courses of osteosarcoma patients with pulmonary metastases who were initially treated in two sarcoma centres in Beijing, China, from June 1st, 2009, to March 26th, 2020. With a median follow-up of 32.4 (95% confidence interval (CI): 30.8, 36.1) months, a total of 127 patients with 605 pulmonary nodules, all of whom had received local therapy and firstly achieved CSR or complete radiated/metabolic remission (CRR), were included in the analysis. A total of 102 patients with 525 nodules were initially diagnosed with resectable lung metastases, while 25 patients had 80 indeterminate nodules at presentation and relapsed with pulmonary metastases within 6 months after the completion of adjuvant chemotherapy.

**Results:**

Eighty-eight of 127 (69.3%) patients had fewer than 5 nodules at the time of local therapy, with 48 of 127 (37.8%) located in the unilateral pleura. No patient underwent thoracotomy, and 42 of 127 patients (85 nodules) received video-assisted thoracoscopic surgery (VATS). In addition, 79 of 127 patients (520 nodules) received hypofractionated stereotactic body radiotherapy (RT), such as Gamma Knife radiosurgery or CyberKnife radiosurgery. The twelve-month event-free survival (EFS) (from local therapy to progression) rate of this entire study cohort was 35.6% (95% CI: 26.8, 44.4%), without a significant difference between the two groups (44.7% for VATS vs. 28.4% for RT, *P* = 0.755). Radiation-induced pneumonitis was observed in 62 of 86 (72.1%) patients, with one patient (1/86, 1.2%) in grade 4.

**Conclusions:**

Our past data showed a similar prognosis with the use of hypofractionated radiotherapy and VATS for the treatment of pulmonary metastasis and no inferiority to thoracotomy regarding historical outcomes. Currently, high-resolution chest computed tomography (CT) provides sufficient information on nodules, and less invasive modalities can thus be considered for treatment.

**Supplementary Information:**

The online version contains supplementary material available at 10.1186/s12885-021-08071-5.

## Background

Patients who develop pulmonary metastasis from osteosarcoma usually have a relatively poor prognosis, with a 5-year overall survival (OS) rate of 20–40%; those who initially present with countable pulmonary lesions have a 5-year OS rate of approximately 40%, whereas those with recurrent osteosarcoma have a 5-year OS rate of 28% or even less [1–3]. The most significant prognosis is the achievement of complete remission [4]. Therefore, the goal of local remission of pulmonary metastases is to render the patient completely disease free. “Tumour debulking” or “cytoreductive surgery” with incomplete resection has not demonstrated any survival benefit. Nevertheless, the timing of local therapy as well as local therapeutic methods remains controversial [5]. To date, thoracotomy has been accepted worldwide for resecting palpable lesions regardless of whether these nodules are present on chest computed tomography (CT) [6, 7]. However, with the development of high-resolution chest CT (with < 3 mm per layer), we aimed to determine whether certain nodules would still be missed on imaging. Can other less invasive local treatment methods replace open resection of pulmonary lesions?

In the last decade, thoracotomy has not been widely performed in China due to numerous reasons. First and most importantly, it has not been realized by the majority of thoracic surgeons that complete surgical remission (CSR) of osteosarcoma plays an important role in its prognosis. Second, although video-assisted thoracoscopic surgery (VATS) has been widely adopted for other pulmonary diseases, it is rarely accepted that doctors perform thoracotomies only for metastatic resection of osteosarcoma. Third, multidisciplinary teamwork (MDT), especially for orthopaedic oncologists, medical oncologists, and thoracic surgeons, is not enough for sarcoma treatment in China. Thus, we rarely perform both orthopaedic and thoracic surgeries together under a single anaesthetic.

Stereotactic body radiotherapy (SBRT) is a theoretically attractive local treatment modality in certain patients with advanced osteosarcoma [8], as it is convenient, minimizes delays in chemotherapy administration, and offers the possibility of increased efficacy via biologically effective dose escalation. Gamma Knife radiosurgery (GKRS) has proven to be a low-risk and effective treatment strategy for a wide variety of patients with brain metastases [9, 10]. In addition, there are a growing number of reports describing the use of an SBRT boost by a robotic stereotactic system (CyberKnife) to an average additional dose of 25 Gy (range: 20–30 Gy) to treat pulmonary lesions in a variety of tumours [11]. Thus, we tried to use GKRS or CyberKnife radiosurgery to treat osteosarcoma pulmonary metastasis in the past. This study aimed to summarize our experience in treating pulmonary metastatic osteosarcoma and compare the outcomes to published historical data abroad to discuss the optimal local therapeutic method for these lesions.

## Methods

From June 1st, 2009, to March 26th, 2020, 973 consecutive patients histologically diagnosed with high-grade osteosarcoma were initially treated at Peking University People’s Hospital (PKUPH) and Peking University Shougang Hospital in Beijing, China. Both hospitals obtained institutional review board approval to review the patients’ medical records as well as radiographic materials. Their outcome data were then retrospectively combined. Written informed consent was not required.

The diagnosis of high-grade osteosarcoma, all established by pathologists using musculoskeletal tumour tissues, was always confirmed and reviewed on histologic slides obtained from resected specimens by two senior pathologists at PKUPH. We included all patients with pulmonary metastasis at the initial presentation, some of whom initially presented with “possible” metastasis according to the EURAMOS-1 protocol [12] and then developed “certain” pulmonary metastasis, with the intention of eradicating all pulmonary nodules. This allowed us to examine the data from 127 patients (605 lesions), all of whom were undergoing surgery or receiving radiation to achieve CSR or complete radiated/metabolic remission (CRR) for the first time.

For high-grade osteosarcomas with pulmonary nodules, we recorded those with the largest diameter (D_max_) of more than 3 mm. However, those with solitary nodules (≥1 cm in diameter) required pathological confirmation by resection, while those with multiple pulmonary nodules (3 mm ≤ D_max_ ≤ 5 mm) were always followed up to confirm their development into metastasis. We recorded the initial time at which these nodules were detected by high-resolution chest CT, which was always assessed by spiral CT scanning, with each layer less than 3 mm. Thoracic surgeons or radiologists always determined whether these pulmonary nodules were resectable based on each individual’s circumstances. Usually, we excluded patients with more than 10 nodules, which might cause severe radiation-induced pneumonitis, from hypofractionated radiotherapy. However, for thoracic surgeries, patients with more than 5 lesions and nodules located at central zones of the lung were also excluded from resection. Specifically, according to radiologists, GKRS was not performed on lesions more than 20 mm in size; however, for CyberKnife radiosurgery, nodule size was not stringently considered a limitation.

The 127 patients enrolled in this study had evident lung metastases based on pathology or long-term follow-up, 102 of whom had certain metastasis at the initial diagnosis, while 25 had indeterminate pulmonary nodules that later developed into certain metastasis. Local therapy was always performed upon effective systemic treatment (i.e., partial response or stable disease based on the RECIST 1.1). However, patients with slow, long-term (6 months or longer) tumour growth were also considered for local eradication. A total of 605 nodules were observed, 85 of which received VATS and 520 of which received RT. With a median follow-up time of 32.4 (95% confidence interval (CI): 30.8, 36.1) months, 110 of 127 (86.6%) patients survived, and 17 of 127 (13.4%) died of disease. Thus, we selected event-free survival (from VATS or RT to any event related to disease progression) rather than overall survival (OS, from VATS or RT to death) to analyse patient outcomes.

During systemic treatment, patients underwent high-resolution chest CT and radiography of the operated musculoskeletal sites every 2 months. After the completion of local therapy, patients were also followed every 2 months for the first 2 years and then every 3 months for the next 3 years. Radionuclide bone scans or positron emission tomography (PET)/CT was used to assess metastatic disease every 6 months for the first 5 years after the completion of systemic treatment.

For this study, event-free survival (EFS) was calculated from the local eradication of pulmonary lesions (resection or radiation) until any event related to disease progression or death, whichever came first. We calculated OS from the time of local therapy to death from any cause. Demographic data are displayed as descriptive statistics. Kaplan-Meier analysis was employed to determine OS, EFS, and recurrence-free survival (RFS). Cox proportional hazards analysis was subsequently performed on variables to identify factors associated with survival and local recurrence. Differences with a *P* value < 0.05 were considered statistically significant. All analyses were performed using SPSS 19.0 software (SPSS Inc., Chicago, IL, USA).

## Results

Patient demographics are summarized in Table 1. At the initial diagnosis, the median age of all eligible patients was 15.0 years (range: 5.0 to 56.0 years). One hundred and fifteen osteosarcomas (90.6%) were situated in the extremities, and 7 osteosarcomas (5.5%) were in the axial skeleton. One osteosarcoma originated from the maxillofacial site, and two originated from the ribs. Eighty-eight of 127 (69.3%) patients had < 5 pulmonary nodules, and 48 of 127 (37.8%) had nodules that were unilaterally located. Based on the D_max_ of the largest pulmonary nodule of each individual treated during local therapy, patients were classified into four categories: 3–5 mm (18/127, 14.2%), 5–10 mm (29/127, 22.8%), 10–20 mm (50/127, 39.4%), and > 20 mm (30/127, 23.6%). A total of 80.3% (102/127) of these patients had synchronous pulmonary metastasis at diagnosis, while 19.7% (25/127) had uncertain pulmonary nodules during the initial diagnosis according to the EURAMOS-1 protocol [12]; additionally, nearly all uncertain pulmonary nodules developed into certain metastases within 6 months after the termination of first-line chemotherapy (Appendix Fig. 1).

**Table 1:** Patient characteristics (*N* = 127)

Items	Number of patients	Percentage (%)	p for 2-y EFS^**a**^
Sex			0.759
Male	83	65.4	
Female	44	34.7	
Age (median: 15.0 years)	Range: 5–56 (Q1, Q3, 15.1, 18.0) years		0.025
< 40 years	124	97.6	
≥ 40 years	3	2.4	
Pathological Subtypes			0.526
Conventional:chondroblastic	12	9.4	
Conventional: osteoblastic	78	61.4	
Conventional: not defined	24	18.9	
Telangiectatic	5	3.9	
Small cell	3	2.4	
High-grade surface	1	0.8	
Missing	4	3.1	
Primary site			0.328
Distal femur	62	48.8	
Proximal tibia and/or fibula	38	29.9	
Proximal femur	4	3.2	
Proximal humerus	9	7.1	
Axial skeleton	7	5.5	
Maxillofacial site	1	0.8	
Others	6	4.7	
Total number of pulmonary nodules for observation	605	100	0.963
Lung metastasis			0.464
≤ 5 nodules	88	69.3	
> 5 nodules	39	30.7	
Lung metastasis			0.063
Monolateral	48	37.8	
Bilateral	79	62.2	
Local therapy for pulmonary nodules			0.476
Resection^b^	42 (85 nodules)	33.1	
Radiotherapy^c^	79 (520 nodules)	62.2	
Combined with resection and radiotherapy	6	4.7	
Missing nodules during follow-up^d^	52	7.9 (52/657)	N/A^i^
For resection	15	15.0 (15/100)	
For radiotherapy	37	6.6 (37/557)	
Failed local resection^e^	8	9.4 (8/85)	
Failed local radiotherapy^f^	14	2.7 (14/520)	
D_max_^g^ for pulmonary nodule/nodules			0.286
3–5 mm	18	14.1	
5–10 mm	29	22.8	
10–20 mm	50	39.4	
> 20 mm	30	23.6	
Systematic treatment during local therapy of pulmonary nodues^h^			0.426
MAPI first-line chemotherapy	56	44.1	
IE second-line chemotherapy	53	41.7	
Targeted therapy	13	10.2	
Combination of TKI_S_ and IE chemo	4	3.2	
None	1	0.8	
Median time for follow-up (months)	32.4	(95% CI: 30.8, 36.1)	(Range: 10.4, 106.5)

^a^2-y EFS: 2-year event-free survival, which was calculated from start of the local therapy (resection or radiotherapy) to any kind of progression as defined by RECIST 1.1

^b^Pulmonary metastasectomies were video-assisted thoracoscopic Surgery (VATS)

^c^Radiotherapy usually involves GammaKnife or Cyber Knife with radio-dose > 60 Gy

^d^By comparing initial chest thin-layer computed tomography (CT) before local therapy and during follow-up, we observed that nodules had resolved or were undetectable with local treatment, most of which were observed as tiny or blurry nodules or even hardly been detected between infection and malignancy and would relapse after stopping systemic treatment

^e^Failed local resection: local tumor relapse where previous tumor resection had been done

^f^Failed local radiotherapy: local tumor relapse where previous radiation had been performed for curative tumor eradication

^g^Patients were classified into four groups based on maximal nodule diameter: 1) 3 mm–5 mm; 2) 5 mm–10 mm; 3) 10 mm–20 mm; 4) > 20 mm

^h^At the Musculoskeletal Tumor Center of Peking University People’s Hospital and Peking University Shougang Hospital, a chemo-protocol that includes high-dose methotrexate, cisplatin, doxorubicin, and ifosfamide (MAPI) is used as first-line chemotherapy (seen in appendix Fig. 1); ifosfamide and etoposide (IE) as second-line systematic therapy; anti-angiogenesis tyrosine kinase inhibitors (TKIs) such as apatinib, anlotinib, cabozantinib, and regorafenib as third-line therapy; the combination of TKIs and IE chemotherapy as fourth-line therapy

^i^Data not available

At the time of local therapy, the median maximum diameter (D_max_) of the treated lesion was 15.8 (IQR, 7.5, 22,4) mm. For radiation, the gross target volume (GTV) was calculated for image-visible lesions based on 1.5-mm slice thickness chest CT, and 70 ~ 85% isodose lines covered the target areas of the planning target volume (PTV). The PTVs were radiated based on the GTVs with reference to the 4D-CT images, and the targets were divided according to the volume and position of the lesions. For CyberKnife radiosurgery, 24/30 Gy/1 f (24 or 30 Gy) was given to lesions that were not close to the mediastinum and chest wall, 45 Gy/3 f was given to lesions close to the chest wall and mediastinum, and 36 Gy/3 f was given to lesions close to the hilum and bronchial tree. Non-isocentre non-coplanar radiation technology was used to design the treatment plan, and stereotactic radiotherapy was performed. A median dose of 26 (IQR, 20, 33) Gy was delivered to these lesions.

Fifty-six of 127 (44.1%) patients underwent tumour resection or received radiation during first-line chemotherapy following the PKUPH-OS regimen (Appendix Fig. 1), which included high-dose methotrexate, doxorubicin, cisplatin, and ifosfamide. Fifty-three of 127 (41.7%) patients were stabilized upon second-line chemotherapy, which included ifosfamide (1.8 g/m^2^/d d_1–5_) and etoposide (100 mg/m^2^/d d_1–5_ Q3W), most of who were initially diagnosed with indeterminate pulmonary nodules. We eradicated metastases in 13 of 127 (10.2%) patients upon stable disease using anti-angiogenetic tyrosine kinase inhibitors (TKIs), such as apatinib and regorafenib, while 4 of 127 (3.2%) received a combination of TKIs and chemotherapy. One patient did not receive any systemic therapy because he had indeterminate pulmonary nodules at presentation and slow tumour growth after the completion of chemotherapy and refused to receive any additional chemotherapy peri-operatively.

In the univariate analysis, we compared sex, age, pathological subtypes, primary tumour site, timing of presentation with pulmonary lesions, nodule number, location in the unilateral pleura, local therapeutic methods, maximum diameter of the lesion treated, and lines of systemic therapy during local therapy, of which only age significantly influenced EFS. However, we analysed only 3 patients older than 40 years; therefore, these results may be inconclusive based on the disparity in the population. After controlling for all these confounders for the multivariate analysis, none remained independent factors associated with EFS, which implied us that the prognosis might not be different only if we got the way to get rid of all the metastatic lesions and tumor number or locations might not influence the local therapeutic efficacy for radiotherapy if we had planned well.

### For the past few years, what kinds of local treatments have most Chinese patients with resectable pulmonary metastasis selected?

Forty-two of 127 (33.1%) patients with 85 nodules chose to undergo resection via VATS. No one had ever received thoracotomy, partly because of the physician’s preference and partly because of a lack of knowledge of thoracotomy or because of fear of trauma. Seventy-nine of 127 (62.2%) patients received RT to eradicate 520 nodules.

### Was there selection bias in choosing between pulmonary metastasectomy and radiation?

A comparison of the clinical and pathological factors of the two groups of patients is presented in Table 2. Forty-one of 42 (97.6%) patients who underwent VATS had < 5 pulmonary nodules, while only 55 of 79 (69.6%) patients who received RT had < 5 pulmonary nodules. In addition, six patients received both RT and VATS. For lesions that was difficult to access during VATS, surgeons marked the lesions on chest CT and advised patients to receive RT 2 weeks after surgery. Patients with bilateral metastasis chose RT more than VATS. VATS was generally chosen by patients with a maximal lesion diameter between 10 mm and 20 mm (24/42, 57.1%). For radiation, patients were almost equally distributed among different size groups. However, it seemed that patients who received VATS had more indeterminate pulmonary nodules at presentation, most of whom needed pathological confirmation through surgery.

**Table 2:** Comparison of clinical manifestations of patients who underwent VATS^a^ or radiation

Items	VATS (***N*** = 42)	Radiotherapy (***N*** = 79)	Combination (***N*** = 6)
Number of pulmonary nodules /person			
≤ 5 nodules	41 (97.6%)	55 (69.6%)	4 (66.7%)
> 5 nodules	1 (2.4%)	24 (30.4%)	2 (33.3%)
Lung metastasis			
Monolateral	33 (78.6%)	11 (13.9%)	4 (66.7%)
Bilateral	9 (21.4%)	68 (86.1%)	2 (33.3%)
D_max_^b^ for pulmonary nodule/nodules			
3–5 mm	3 (7.1%)	15 (19.0%)	0 (0.0%)
5–10 mm	6 (14.3%)	22 (27.8%)	1 (16.7%)
10–20 mm	24 (57.1%)	25 (31.6%)	1 (16.7%)
> 20 mm	9 (21.4%)	17 (21.5%)	4 (66.7%)
Systematic treatment during local therapy of pulmonary nodues^c^			
MAPI first-line chemotherapy	14 (33.3%)	40 (50.6%)	2 (33.3%)
IE second-line chemotherapy	24 (57.1%)	25 (31.6%)	4 (66.7%)
Targeted therapy	2 (4.8%)	11 (13.9%)	0 (0.0%)
Combination of TKIS and IE chemo	2 (4.8%)	4 (5.1%)	0 (0.0%)
None	0 (0.0%)	1 (1.3%)	0 (0.0%)

^a^*VATS* Video-assisted thoracoscopic surgery

^b^Patients were classified into four groups based on nodule maximal diameter: 1) 3 mm–5 mm; 2) 5 mm–10 mm; 3) 10 mm–20 mm; 4) > 20 mm

^c^At the Musculoskeletal Tumor Center of Peking University People’s Hospital and Peking University Shougang Hospital, a chemo-protocol that includes high-dose methotrexate, cisplatin, doxorubicin, and ifosfamide (MAPI) is used as first-line chemotherapy (seen in appendix Fig. 1); ifosfamide and etoposide (IE) as second-line systematic therapy; anti-angiogenesis tyrosine kinase inhibitors (TKIs) such as apatinib, anlotinib, cabozantinib, and regorafenib as third-line therapy; and the combination of TKIs and IE chemotherapy as fourth-line therapy

### Did different local therapy methods influence patient outcomes?

**Fig. 1:**
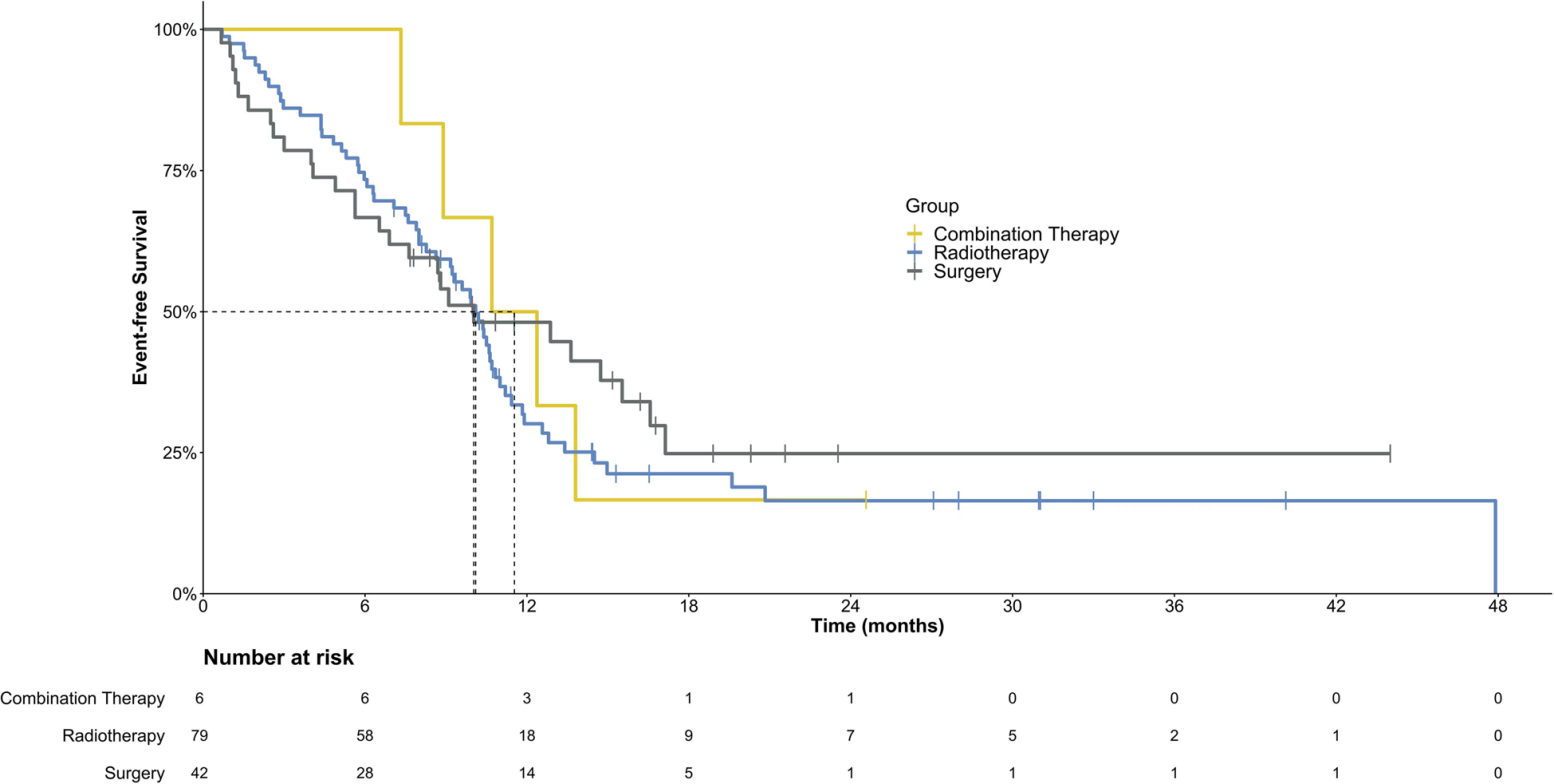
Kaplan-Meier plot for event-free survival in all 127 patients

**Fig. 2:**
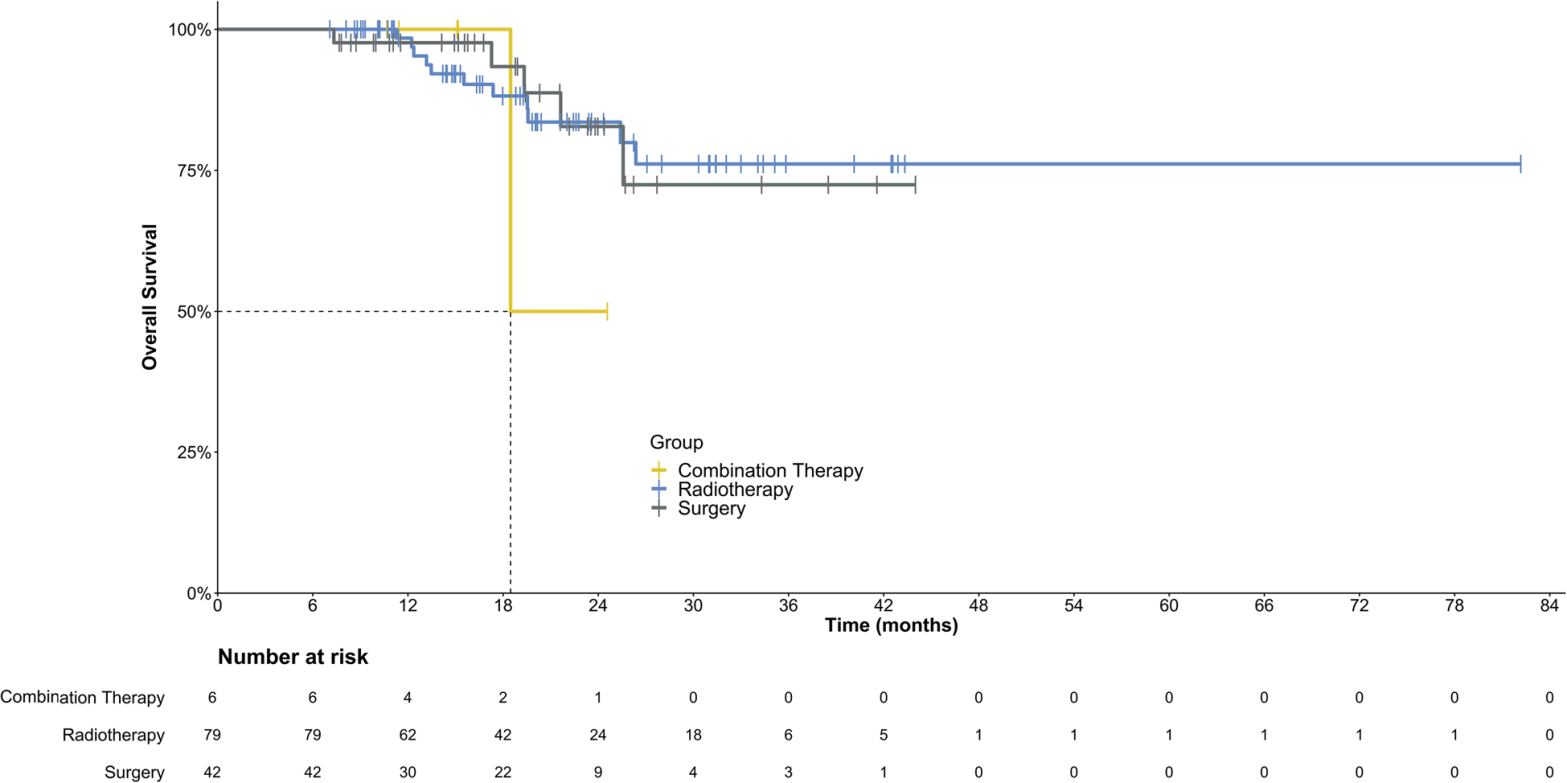
Kaplan-Meier plot for event-free survival based on different local treatment methods. (1 indicates patients who received radiotherapy; 2 indicates patients who underwent surgery; and 3 indicates patients who received a combination of radiotherapy and surgery). Log-rank test *P* = 0.755. Crosses indicate censoring

Until the last follow-up, local relapse after VATS occurred in 9.4% (8/85) of patients, while local recurrence after RT occurred in 2.7% (14/520) of patients (Table 3). The 2-year non-local recurrence survival rates [SD] were 81.0% [7.1%] and 92.8% [3.1%] for resection and radiation, respectively (*P* = 0.37). The median EFS times were 10.0 (IQR, 4.1, 17.1) and 10.1 (IQR, 5.8, 14.5) months for VATS and RT, respectively, without a significant difference (*P* = 0.76) (Figs. 1 and 2).

**Table 3:** Comparison of survival in different groups of patients

Items	Patients with Resections (***N*** = 42)	Patients with Radiotherapy (***N*** = 79)	Patients with combination of resections and radiotherapy (***N*** = 6)	p for survival
2-year no local recurrence survival rate [±SD]	81.0% [±7.1%]	92.8% [±3.1%]	66.7%[±36.7%]	0.652
Local relapse of nodules without new lesions	2/42 (4.8%)	1/79 (1.3%)	1/6 (16.7%)	N/A^a^
Local relapse of nodules with new lesions	6/42 (14.3%)	10/79 (12.7%)	1/6 (16.7%)	N/A^a^
Progression without local relapse	19/42 (45.2%)	49/79 (62.0%)	3/6 (50.0%)	
Events for progression in total	27/42 (64.3%)	60/79 (75.9%)	5/6 (83.3%)	
From resections/radiotherapy to any event (median, Q1, Q3) months	10.0 (4.1, 17.1)	10.1 (5.8, 14.5)	N/A	0.755
From resections/radiotherapy to death (mean, 95%CI)^b^ months	37.6 (32.5, 42.7)	67.0 (58.7, 75.3)	21.5 (17.3, 25.7)	0.712

^a^N/A data not available

^b^median overall survival has not reached yet, thus we use mean overall survival to replace the data

**Fig. 3:**
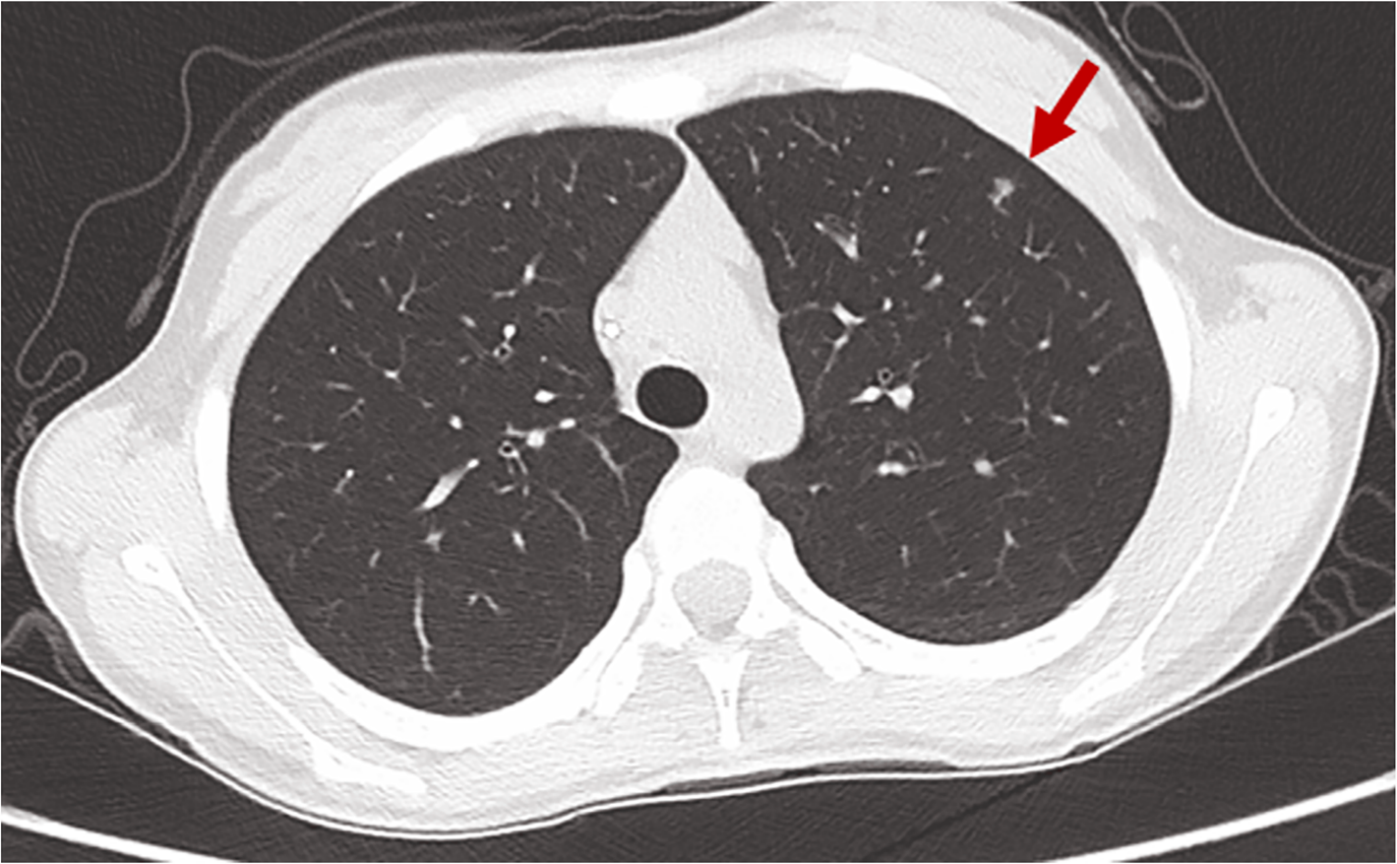
A missing nodule shown on chest CT (red arrow) before metastasectomy. (It was not resected later. CT = computed tomography)

**Fig. 4:**
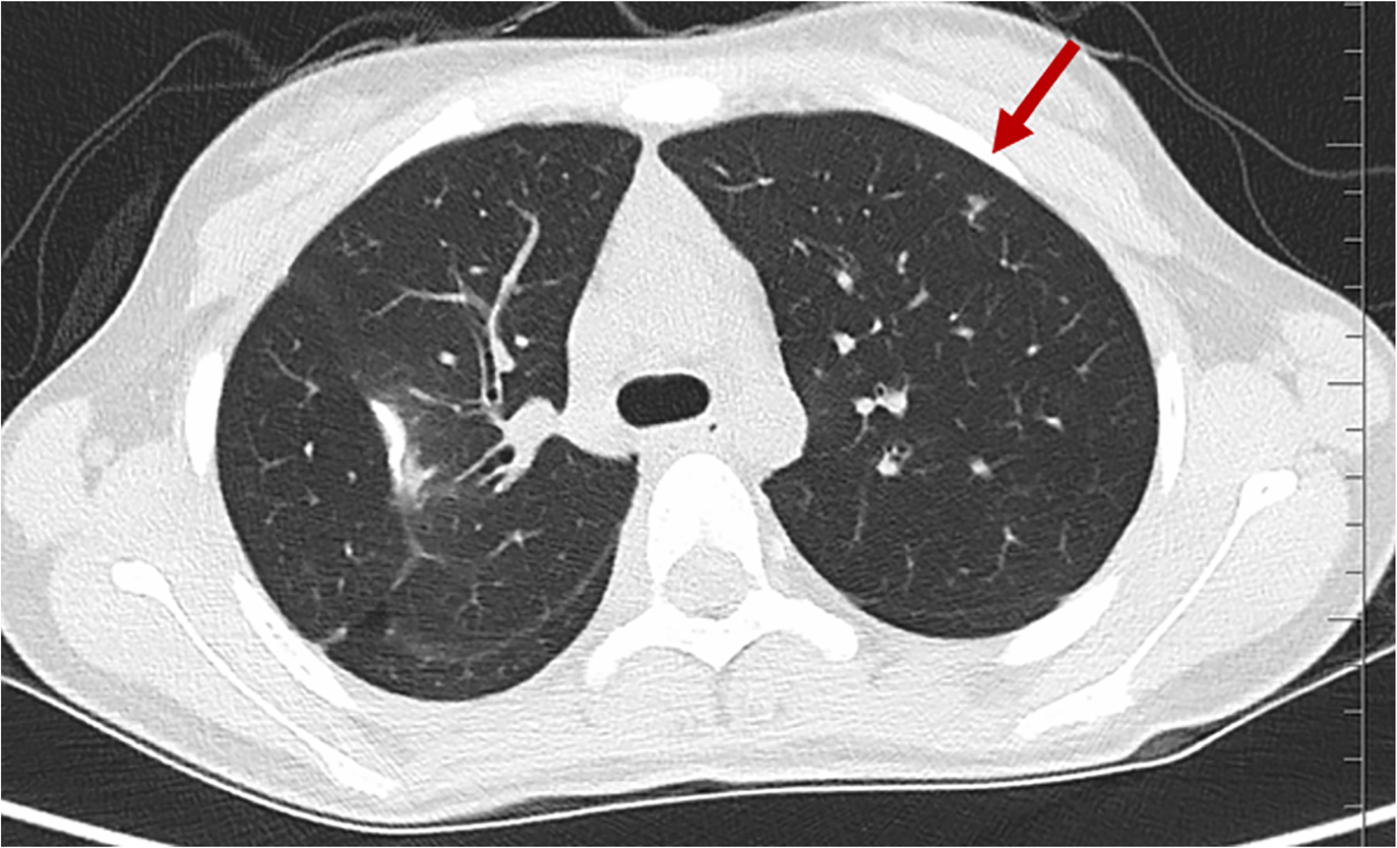
A missing nodule shown on chest CT (red arrow) two weeks after metastasectomy. (A surgical scar can be observed on the right lobe of the lung. CT = computed tomography)

**Fig. 5:**
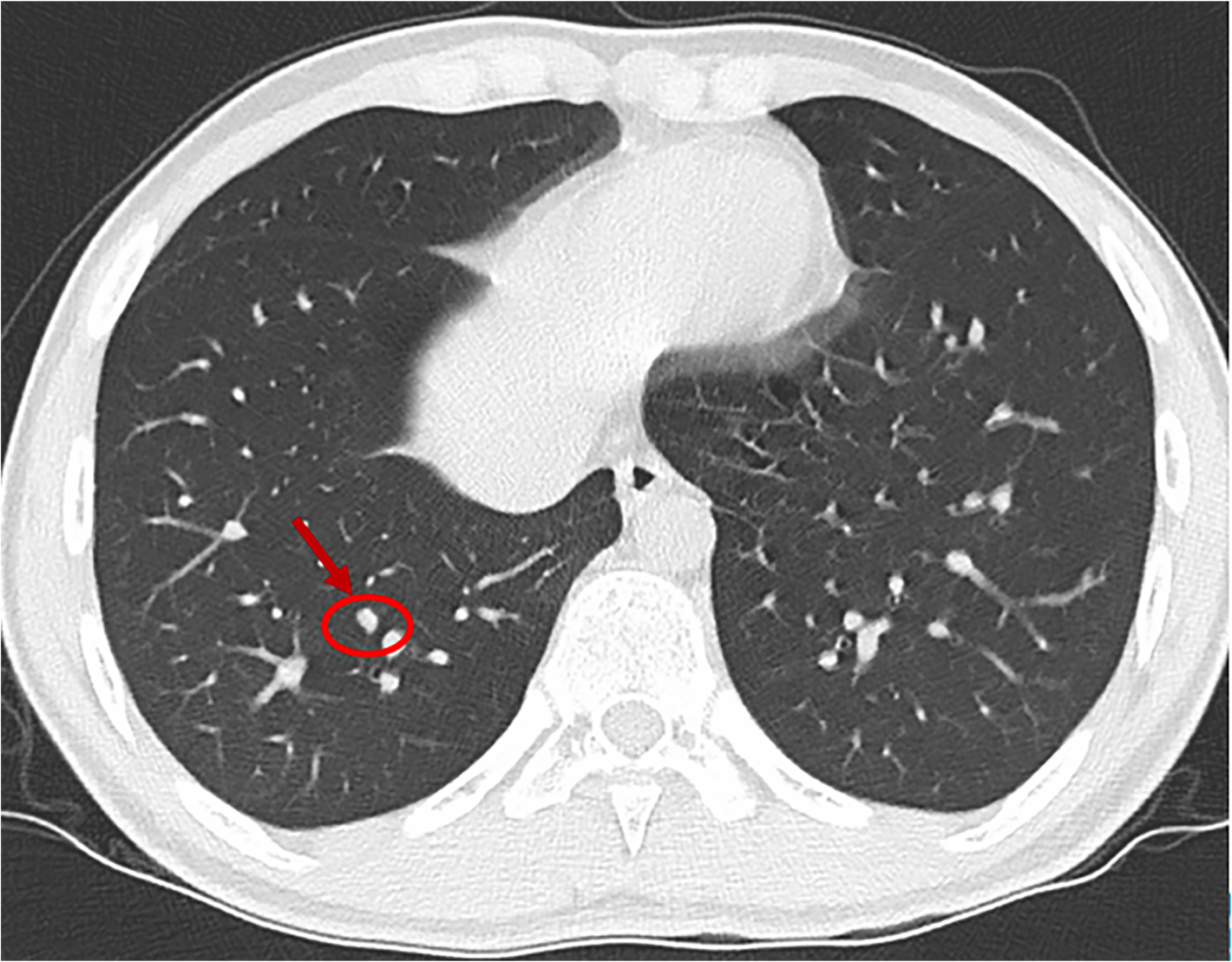
A nodule less than 5 mm missing on chest CT (red circle) for radiation (This image was taken before hypofractionated radiotherapy. CT = computed tomography)

**Fig. 6:**
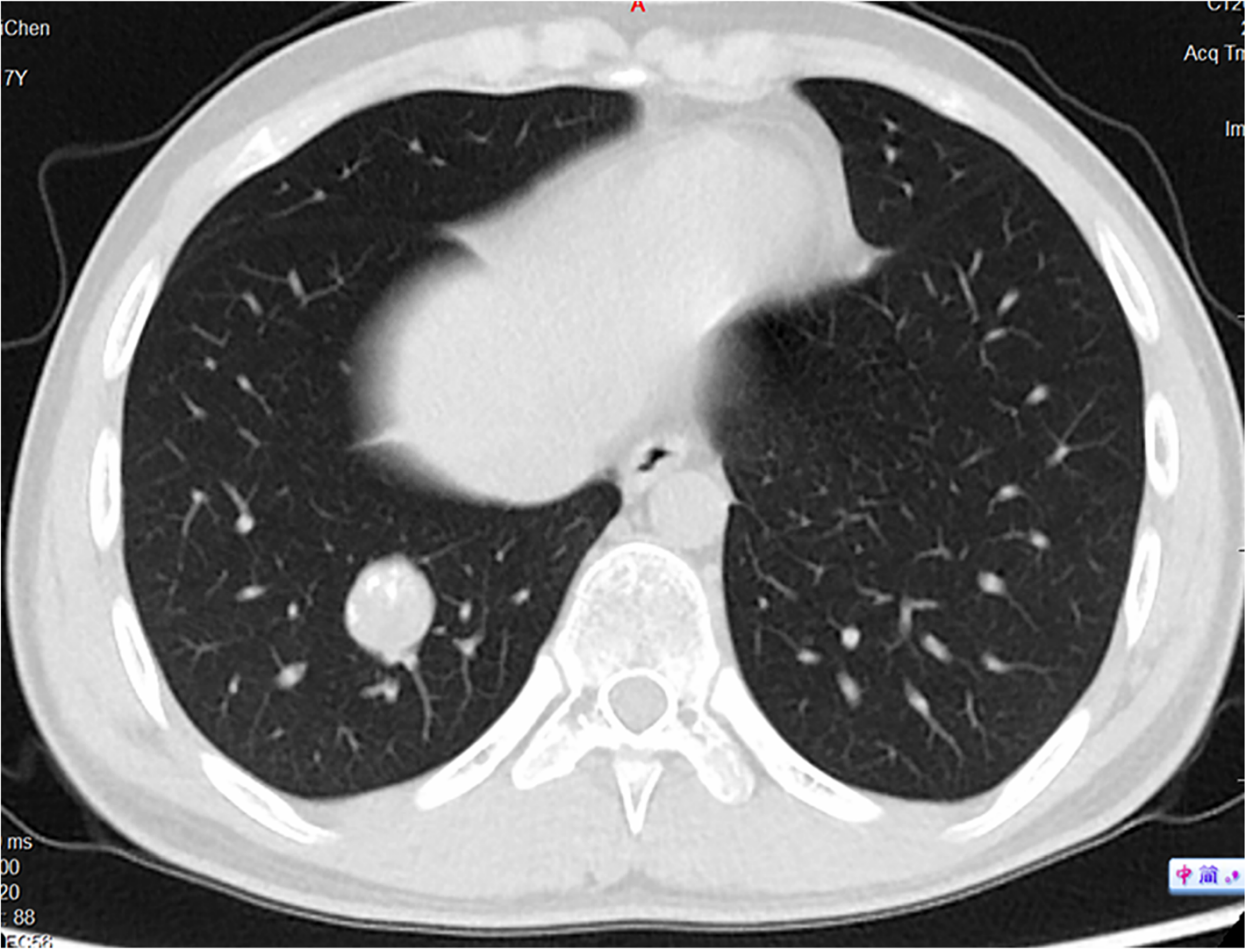
A missing nodule progressed on chest CT (red circle) after radiation. (Eleven months later, this nodule, which had been missed for local therapy, became larger and obvious, and 3 new lesions also appeared on this patient’s chest CT, all of which were later subjected to a second cycle of radiotherapy. At the last follow-up, the patient was still disease free. CT = computed tomography)

A review of all these high-resolution chest CT scans revealed that numerous nodules were missed (Figs. 3, 4, 5 and 6) before local therapy regardless of whether the patients received VATS or RT. For 85 resected nodules, high-resolution chest CT 2 weeks after surgery (we usually performed a scan shortly after surgery in the case of pneumothorax or haemothorax to deliver postoperative systemic therapy) showed 15 nodules missing (17.7%, all < 5 mm) that later grew larger in size and were confirmed as metastasis clinically or pathologically. For radiation, by comparing the chest CT scan before RT with target-planning photographs for RT, we noticed that 7.12% (37/520) of the nodules were missed for eradication and later progressed. Detailed information is presented in Table 3. In addition, we observed that the majority of cases of disease progression were not accompanied by local relapse. No correlation between local relapse and disease progression was detected (*P* = 0.37).

### How do the data of the present study compare to those of published historical controls?

In total, these patients showed a 12-month EFS rate of 35.6% (95% CI: 26.8, 44.4%), which was concordant with the findings of the Children’s Oncology Group (20 to 31% in the prospective trials of AOST1221 [13] and AOST1421 [14]). For patients who received VATS, the 12-month EFS rate was 44.7%, whereas for patients who received RT, it was 28.4%; however, this difference was not statistically significant (*P* = 0.76, Fig. 2). Our findings on OS should be considered preliminary. No obvious difference in prognosis was observed between the patients in our study and those who underwent open resection abroad. Our 2-year EFS rate was estimated to be 18.6% based on Kaplan-Meier analysis, which was not inferior to the Cooperative Osteosarcoma Study Group (COSS) results on recurrent osteosarcoma [12].

### What was the prevalence of radiation-induced pneumonitis in these patients?

At follow-up, 62 of 86 (72.1%) patients presented with pneumonitis on chest CT after radiation, 8 of whom (9.3%) developed grade 2 pneumonitis according to the Common Terminology Criteria for Adverse Events (CTCAE 5.0) and required steroids for palliation. One patient had pneumonitis that was so severe that she was administered steroid-included therapy for more than 3 months and later developed *Pseudomonas aeruginosa* infection. She ultimately died of disease progression or pyothorax (the data were not clear). The median time to the development of pneumonitis in our study was 3.5 (95% CI: 1.0, 5.5) months after RT, with a duration of 7.2 (95% CI: 6.9, 12.3) months (detailed information is presented in Table 4).

**Table 4:** The incidence and duration of radiation-related pneumonitis (*N* = 86)

Items	Number of patients	Percentage
Pneumonitis Grade^a^	62	72.1%
Grade 1	53	61.6%
Grade 2	8	9.3%
Grade 4	1	1.16%
Onset of pneumonitis (Median, 95% CI)	3.5 (1.1, 5.5) months	
Duration of pneumonitis (Median, 95% CI)	7.2 (6.9, 12.3) months	
Pneumonitis still not resolved until last follow up	20	23.3%

^a^According to CTCAE 5.0

## Discussion

This retrospective study showed that in China, the EFS rate for osteosarcoma patients with resectable pulmonary metastasis who received hypofractionated RT was comparable to that for those who received VATS during the same period and for those who underwent thoracotomies in published historical controls. The 12-month EFS rate in our total population was 35.6% (95% CI: 26.8, 44.4%), with 44.7% for VATS and 28.4% for radiation (*P* = 0.76). The COG conducted two prospective trials using small cohorts of patients with pulmonary recurrent osteosarcoma who received maintenance immunotherapy within 4 weeks after surgical complete remission, of which the 12-month EFS rates were 20% (95% CI: 10, 34%) and 31% (95% CI: 17, 45%), respectively, without a significant improvement in prognosis [13, 14]. The COSS also previously evaluated the impact of patient-, tumour-, and treatment-related factors on the outcome in unselected patients with recurrent osteosarcoma recruited from a series of COSS studies between the end of 1979 and July 1998 [12]. At a median follow-up of 1.2 years for all patients and 4.2 years for survivors, the actuarial OS rates at 2, 5, and 10 years were 0.38, 0.23, and 0.18, respectively, which, in our opinion, is slightly low, especially in terms of 2-year OS. This might be due to the lack of more effective therapeutic schemes that were executed 30–40 years ago. Our actual OS data are preliminary; thus, they were not comparable. However, from the Kaplan-Meier EFS curves of the COSS, we observed that the 2-year EFS rate was 19% (104/576) since relapse. Our data showed that the 2-year EFS rates were 15.4 and 10.9% for RT and VATS, respectively, indicating no inferiority to the COSS outcomes. Rizzoli et al. [6, 7, 15] conducted a series of studies that revealed the prognosis for high-grade osteosarcoma of the extremities metastatic to the lung, with a 5-year EFS rate of 36% for patients with localized disease who later relapsed and 9% for patients with resectable lung metastases at presentation; the difference was highly significant (*P* < 0.0001). However, they calculated EFS from the beginning of treatment to relapse. It is interesting to note that in our study, the 5-year EFS rate was 18.6% for patients with initial metastasis (Kaplan-Meier estimated, when calculated from starting treatment to any event), which also showed no inferiority.

Sex, location, pathological subtypes, the number of pulmonary nodules, location at the unilateral pleura, local therapeutic methods, and lines of systemic therapy were irrelevant to prognosis in our study (Table 1). However, in our study, 3 of 127 (2.4%) patients were older than 40 years and showed poorer EFS than those under 40 years (*P* = 0.025). Cox multivariate analysis indicated that no factor was correlated with outcomes. Thus, we hypothesize that currently, high-resolution chest CT (< 3 mm per layer) can provide sufficient information for tiny pulmonary nodules to eradicate all lesions before RT or surgery, similar to thoracotomies [4, 5, 7, 12, 15]. However, we observed that 7.9% (52/657) of nodules were missed during follow-up; of which, for resection and for radiation, the missing rates were 15.0% (15/100) and 6.6% (37/557), respectively. Table 3 also shows that only 1.3–4.8% of these untreated nodules alone would cause disease progression, while 12.7–14.3% would relapse together with multiple new lesions, which indicated that these poor events were due to the progression of systemic disease and not to the untreated lesions. Furthermore, we observed that 45.2–62.0% of the cases of progression were not coupled with the local relapse of any existing lesion. Thus, we infer that with a local relapse rate as low as 5–15%, disease progression is not because of local recurrence of pulmonary nodules, and such kinds of local therapy methods are adoptable.

However, radiation-induced lung injury (RILI), especially acute radiation pneumonitis, was observed in the majority of patients (72.1%), with a median onset time of 3.5 (95% CI: 1.1, 5.5) months and a duration of 7.2 (95% CI: 6.9, 12.3) months. For cases involving > 10 lesions that were larger than 4 cm and located beside the major bronchi, pneumonitis was so severe that we usually administered steroids to palliate symptoms. However, for RILI, the duration of corticosteroid use should always be 4 weeks or longer, which often causes a disruption in the anti-tumour immune microenvironment as well as secondary infections. Thus, currently, we choose VATS combined with RT for more complicated cases. Radiation is indicated for lesions located in sites that are difficult to manage during VATS. Surgeons often mark the chest CT scan 2 weeks after surgery for RT.

### Limitations

This study had some limitations, including its small sample size and retrospective design. Selection bias was unavoidable in choosing patients who would receive local therapy. During the period in question, we had some general indications for the choice of surgery or radiation, as mentioned above. However, as a retrospective study, we are not certain that these indications were rigorously adhered to. The choice of local therapeutic method was deeply impacted by the patients’ preferences. During our past experience, patients who had solitary lesions or less than 3 lesions and needed pathological confirmation conventionally received surgery, while those with more than 3 lesions or pulmonary nodules located in the central zone of the lung, which were deemed difficult to resect by VATS, usually received radiotherapy instead. Furthermore, the more times patients experienced pulmonary lesion relapse, the more the patients were inclined to choose radiation. Second, our follow-up time was not long enough to demonstrate OS and compare OS with that of historic controls. Third, we did not record in detail patients with secondary complete remission of pulmonary lesions or compare the outcomes with those who underwent CSR or CRR for the first time. Fourth, as only two Chinese sarcoma centres were involved in this study, our data might not completely reflect the current situation of osteosarcoma treatment in China. However, the local treatment of these pulmonary lesions was performed between June 2014 and July 2020 and in only two centres that might have used consistent systemic therapy and local therapy methods. Our experience should be shared and discussed when assessing local treatment options for such patients.

## Conclusions

In patients with recurrent resectable pulmonary metastases from high-grade osteosarcoma that were deemed stable under systemic therapy, both hypofractionated RT and VATS can be considered adoptable options for local therapy methods because high-resolution chest CT can provide sufficient information on all these nodules. Therefore, the missing rate for these nodules could be relatively low, and we could mark and eliminate all the nodules. However, extra caution should be taken when using radiation to avoid severe pneumonitis.

## Supplementary Information

**Additional file 1 Appendix Fig. 1** Pictured is the updated osteosarcoma protocol used at Peking University People’s Hospital-Osteosarcoma (PKUPH-OS 02). DOX = doxorubicin; CDDP = cisplatin; IFO = ifosfamide; VCR = vincristine; HD MTX = high-dose methotrexate.

## Data Availability

We promised to maintain patient data confidentially. However, patient data will be made disguisedly available (including data dictionaries) for approved data sharing requests. Individual data that underlie the results reported in this article will be shared after de-identification and normalization of information (text, tables, figures, and appendices). The statistical analysis plan will also be available upon request. Anonymised data will be available beginning 3 months after and ending 2 years after publication of this article to researchers after a methodological review of the proposed analysis plan by the directors of the Musculoskeletal Tumor Center of Peking University People’s Hospital or Peking University Shougang Hospital. Proposals should be directed to xie.lu@hotmail.com. To gain access, data requestors will need to sign a data access agreement.

## Abbreviations

RT: Radiotherapy
OS: Overall survival
CT: Computed tomography
CSR: Complete surgical remission
VATS: Video-assisted thoracoscopic surgery
MDT: Multidisciplinary teamwork
SBRT: Stereotactic body radiotherapy
GKRS: Gamma Knife radiosurgery
PKUPH: Peking University People’s Hospital
CRR: Complete radiated/metabolic remission
CI: Confidence interval.
PET: Positron emission tomography
EFS: Event-free survival
TKIs: Tyrosine kinase inhibitors
AOST: Osteosarcoma Protocols from Children’s Oncology Group
COSS: Cooperative Osteosarcoma Study Group
CTCAE: Common Terminology Criteria for Adverse Events
RILI: Radiation-induced lung injury
