# Supplementary figures and images for: Complete remission of metastatic osteosarcoma using combined modality therapy: a retrospective analysis of unselected patients in China
Source: BMC Cancer. 2021 Mar 31;21:337. doi: 10.1186/s12885-021-08071-5 (PMC8010982; doi:10.1186/s12885-021-08071-5)

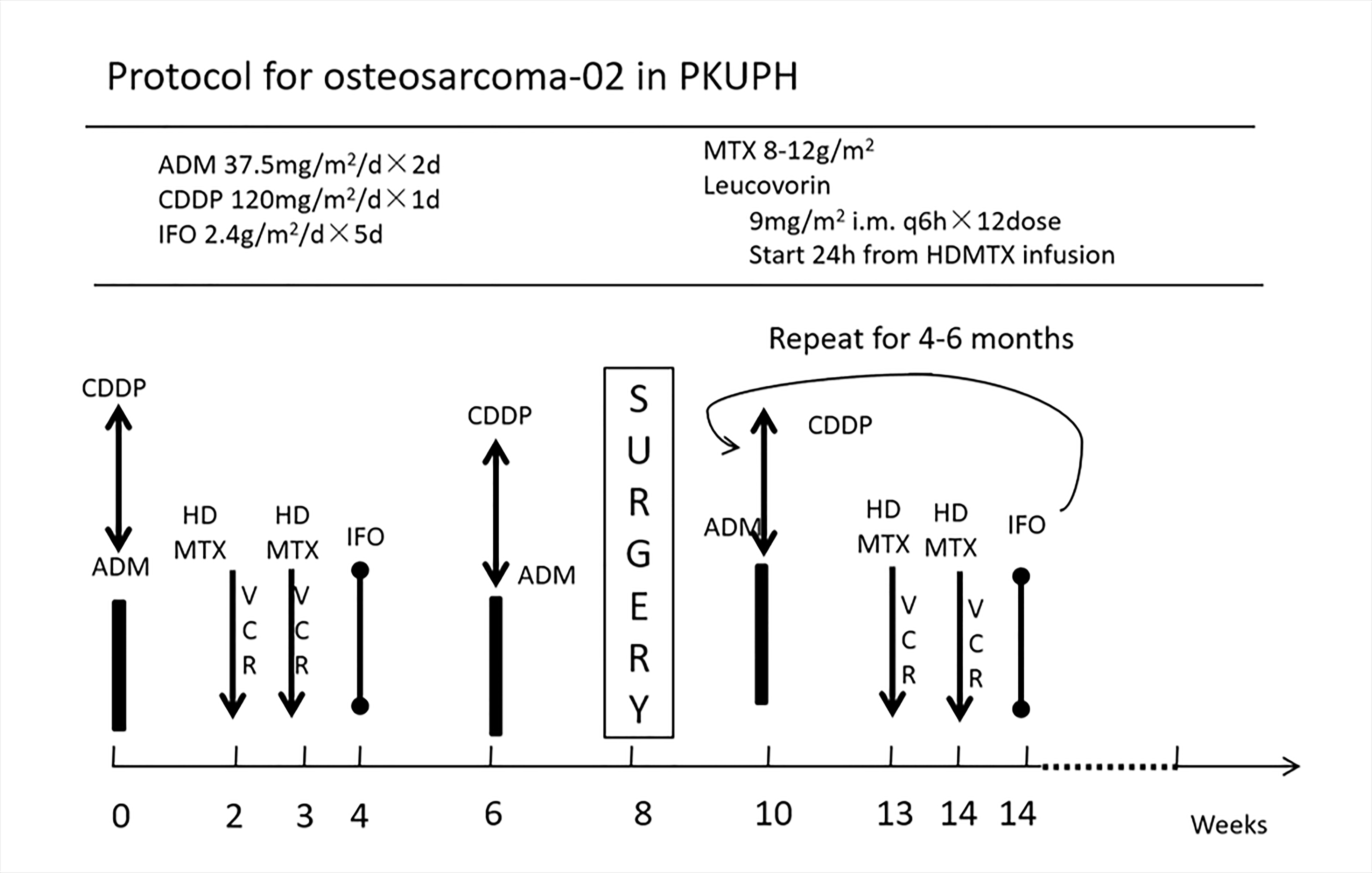

Supplement: Supplementary file 1 — Additional file 1 Appendix Fig. 1 Pictured is the updated osteosarcoma protocol used at Peking University People’s Hospital-Osteosarcoma (PKUPH-OS 02). DOX = doxorubicin; CDDP = cisplatin; IFO = ifosfamide; VCR = vincristine; HD MTX = high-dose methotrexate. [file 12885_2021_8071_MOESM1_ESM.tif]
